# Surgical treatment of hip dysplasia in cerebral palsy: A retrospective comparison between open and closed reduction

**DOI:** 10.1097/MD.0000000000044245

**Published:** 2025-09-05

**Authors:** Marcio Vieira Sanches Silva, Bárbara Lívia Corrêa Serafim, Luiz Renato Agrizzi de Angeli, Alexandre Zuccon

**Affiliations:** a Department of Orthopedics and Traumatology, Santa Rita Hospital, Vitoria, ES, Brazil; b Department of Orthopedics and Traumatology, Brazilian Association for the Welfare of the Handicapped Children (AACD), São Paulo, SP, Brazil; c Institute of Orthopedics and Traumatology, University of São Paulo Medical School, São Paulo, SP, Brazil.

**Keywords:** cerebral palsy, closed reduction, hip dislocation, hip dysplasia, open reduction

## Abstract

Hip dysplasia in cerebral palsy (CP) is a common and severe problem, especially among nonambulatory patients. A likely cause is muscular imbalance and developmental bone changes leading to a progressive extrusion of the femoral head from the acetabulum. The ideal surgical treatment aims to reduce the dislocated hip to improve pain, positioning, and function. The purpose of this study is to clinically and radiographically compare the results of hip reconstructive surgery with or without open reduction in patients with CP and hip dysplasia. A retrospective study was conducted through access to medical records, radiographs, and clinical evaluation of patients with CP who underwent surgical correction of hip dysplasia through hip reconstructive surgery with a minimum of 2 years follow-up. Two surgical techniques were compared: open versus closed reduction. Clinical parameters such as pain, hip abduction, age, follow-up time, and the Caregiver Priorities and Child Health Index of Life with Disabilities Questionnaire were used. Radiographic evaluation included dislocation degree (hip status), Reimers migration percentage, and the presence or absence of osteonecrosis. One hundred thirty hips were included in the study, and 23.08% of patients underwent bilateral procedures. Thirty-three percent of patients were classified as gross motor function classification system IV and 67% were gross motor function classification system V. The mean preoperative reimers migration percentage decreased from 77.4% to 7.6% in the open reduction group and from 76.0% to 6.2% in the closed group (*P* = .656). Postoperative hip abduction increased from 10° to 25° in the open group and from 13.2° to 24.2° in the closed group (*P* = .096). Caregiver Priorities and Child Health Index of Life with Disabilities Questionnaire scores showed no significant differences in Sections II, III, and VII. Osteonecrosis occured in 50% of the open group and 45.3% of the closed group (*P* = .659). Both techniques were effective and had similar clinical and radiographic outcomes in treating hip dysplasia in patients with CP. Further prospective studies are necessary to clarify the best indications for each technique.

## 1. Introduction

Cerebral palsy (CP) is a group of permanent movement and postural disorders resulting from a nonprogressive brain lesion that occurs in early development^[1]^ Hip dysplasia is a serious complication in patients with CP, especially in nonambulatory patients, with a prevalence as high as 80%.^[2,3]^ Spasticity of the hip adductors and flexors, which are stronger than the hip extensors and abductors could cause an imbalance of muscle forces leading to progressive extrusion of the femoral head from the acetabulum.^[4–6]^

Progressive hip dysplasia can cause pain, contractures, difficulty with hygiene and positioning, pelvic tilt, scoliosis, skin ulcers and fractures, among other complications.^[7]^ Attempts to prevent hip dislocation/subluxation include the use of orthotics to keep the hip reduced, physical therapy, botulinum toxin and phenol injections, and surgery to equalize muscle forces with or without proximal femur-guided growth.^[2,8–10]^ Surgical treatment in an advanced subluxation/dislocation stage includes balancing of muscle forces and reduction of the hip by femoral varus derotation osteotomies (VDRO) and acetabular osteotomies. These procedures, known as hip reconstructive surgeries (HRS), can be performed with an open or closed reduction of the hip; however, the optimal approach for reduction remains unclear.^[6,11]^ Existing literature lacks consensus on whether open reduction improves outcomes compared to closed techniques, especially in severe cases.

This study aimed to clinically and radiographically compare outcomes of HRS with or without open reduction in patients with CP. We hypothesized that there would be no significant difference in clinical or radiographic outcomes between the techniques.

## 2. Materials and methods

After approval by the ethics committee from the Brazilian Association for the Welfare of the Handicapped Children, we conducted a retrospective review of patients with CP who underwent HRS between 2000 and 2011. We applied the following inclusion criteria: children with a diagnosis of CP who underwent HRS; gross motor function classification system (GMFCS) IV or V; minimum follow-up of 2 years. Exclusion criteria were: incomplete data; loss of follow-up; use of another surgical technique to treat hip dislocations or subluxations. Informed consent was not required as this was a retrospective study based solely on the review of medical records.

We separated the hips into 2 groups: open and closed reduction. The decision to perform each technique was made intraoperatively based on the surgeon’s assessment of femoral head reducibility and hip stability. Primary outcomes were the postoperative Reimers migration percentage (RMP) and hip abduction. Secondary outcomes included pain, Caregiver Priorities and Child Health Index of Life with Disabilities Questionnaire (CPCHILD) scores ^[12]^, and osteonecrosis rates.

Thus, the following variables were evaluated: age at the time of surgery, postoperative follow-up time, sex, GMFCS, preoperative and postoperative RMP, hip status, which evaluates the severity of dislocation preoperatively based on Tönnis classification (Table S1, Supplemental Digital Content, https://links.lww.com/MD/P829),^[13]^ abduction range-of-motion pre and postoperative, development of osteonecrosis, Visual analog scale (VAS) for pain,^[14]^ and CPCHILD.^[12]^

The CPCHILD is a questionnaire about the quality of life of patients with CP, translated and validated into Portuguese. It was administered to the parents or guardians of the patient during the last consultation. Sections II (difficulty level regarding positioning, transfer, and mobility), III (frequency of pain and discomfort), and VII (importance of daily activities in the patient’s quality of life) were evaluated.^[12]^ The surgical procedures were performed by the pediatric orthopedic surgeons of the institution.

All patients underwent VDRO and a Dega pelvic osteotomy.

For statistical analysis, a significance level of 5% and a confidence interval of 95% were assumed. Statistical tests such as ANOVA, *t* student test, and equality of proportions test were applied to compare the obtained data. Software used included SPSS V17, Minitab 16, and Excel Office 2016. Clinical and radiographic parameters between open and closed reduction were compared and results were reported with means and standard deviations.

### 2.1. Surgical techniques

#### 2.1.1. Open reduction

The adductors and psoas are first released through a medial approach below the inguinal fold. After this initial procedure, the anterior Smith–Petersen approach is performed as standard,^[15]^ with or without preservation of the rectus femoris. The joint capsule is exposed and its surface must be largely freed from the adjacent adipose tissue. The capsular incision is made through a T-shaped incision. The ligamentum teres and the pulvinar are resected if they are thickened, and the transverse acetabular ligament is cut. After these obstacles are removed, we perform the VDRO through a lateral incision in a standard fashion. Following the VDRO, a Dega pelvic osteotomy is performed. Finally, a capsuloplasty is performed and the wounds are closed in a standard fashion.

#### 2.1.2. Closed reduction

**Figure 1.**
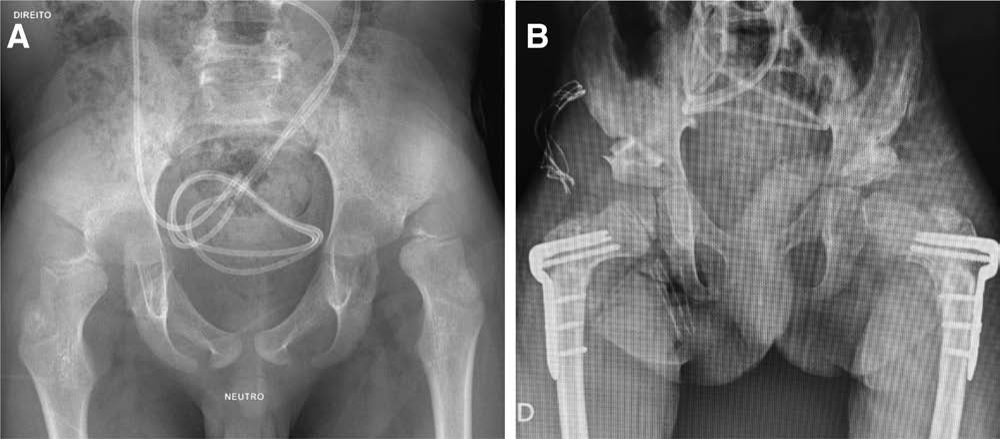
Preoperative (A) and postoperative (B) radiographs of an 8-yr-old GMFCS V boy who was submitted to a bilateral varus derotational osteotomy and a pelvic Dega osteotomy on both sides. Bilateral hip clinical stability was obtained after a closed reduction on both sides. Final Reimers Migration Percentage was 0% bilaterally. (Images from the personal files of the authors). GMFCS = gross motor function classification system.

In closed reduction, the anterior Smith–Petersen approach is not required, therefore, the entire first part of the procedure mentioned for open reduction is not required. In this technique, only the initially mentioned tendinous release of adductors and psoas together with the VDRO are performed, and an additional Dega pelvic osteotomy is performed through an accessory approach. The wounds are then closed in a standard fashion (Fig. 1).

## 3. Results

Out of the 271 patients identified, 60 did not attend the final interview, leaving 211 patients. Of these, 103 had incomplete data. Additionally, 3 patients underwent different techniques from those studied in this investigation and were excluded. Finally, 105 patients (130 hips) were included.

Of the total of 130 hips analyzed, 58 (44.61%) underwent open reduction and 72 (55.39%) underwent closed reduction. Bilateral procedures were performed in 25 (23.08%) patients.

There were 73 (69.52%) male patients and 32 (30.47%) female patients. Among these, 35 (33.33%) were classified as GMFCS IV, and 70 (66.66%) as GMFCS V. The minimum age at the time of the surgical procedure was 6 years, the maximum was 224 months (18.66 years), and the mean age was 116.9 months (9.74 years).

The mean age at the time of surgery in the open reduction group was 116.1 months (76–170), while for those undergoing closed reduction, it was 117.8 months (72–224), showing no significant difference between the 2 groups (*P* = .709). The mean postoperative follow-up time was 49.6 months (24–109) in the open reduction group and 50.6 months (76–170) in the closed reduction group, also showing no difference between samples. When analyzing the RMP in the open reduction group, the mean values dropped from 77.4% to 7.6% after HRS. In the closed reduction group, the average RMP dropped from 76% to 6.2% after surgery (*P* = .656). Table 1 shows the surgical outcome assessment metrics.

**Table 1:** Surgical outcome assessment metrics.

		Mean	Median	Standard deviation	Min	Max	N	CI	*P*-value
Age at surgery	Open	116.1	113	25.7	76	170	28	9.5	.791
	Closed	117.8	119	29.7	72	224	96	5.9	
Post operative follow-up	Open	49.6	45.5	21.0	20	109	28	7.8	.830
	Closed	50.6	47	20.4	24	108	97	4.1	
Reimers before	Open	77.4	100	31.0	37	100	29	11.3	.801
	Closed	76.0	83	25.6	33	100	95	5.1	
Reimers after	Open	7.6	0	15.5	0	66	28	5.8	.656
	Closed	6.2	0	14.6	0	100	96	2.9	
Abduction before	Open	10.2	10	7.1	0	30	28	2.6	.096
	Closed	13.2	10	8.9	0	45	97	1.8	
Abduction after	Open	25.2	20	14.0	10	90	29	5.1	.702
	Closed	24.2	20	11.7	0	90	97	2.3	
Pain VAS after	Open	1.4	0	2.5	0	8	25	1.0	.371
	Closed	0.9	0	2.3	0	9	94	0.5	

All values are expressed in months (age and follow-up), percentages (Reimers), degrees (abduction), or points (VAS).

CI = confidence interval, min = minimal, max = maximal, N = sample size, VAS = visual analog scale.

The preoperative mean clinical hip abduction measurement of patients undergoing closed reduction rose from 13.2° (0–45°) to 24.2° (0–90°) after HRS. For patients undergoing open reduction, mean values increased from 10° (0–30°) to 25° (10–90°) after surgery. There were no significant differences between groups (*P* = .096).

VAS for pain, collected in the last consultation, was 1.4 for patients who underwent open reduction compared to 0.9 for patients who underwent closed reduction (*P* = .371), as shown in Table 1. Both ratings fall within the range described as painless or mild pain.

The CPCHILD questionnaire^[12]^ revealed a score of 10.7 in Section II, 40.4 in Section III, and 154.7 in Section VII for patients who underwent a closed reduction procedure. Patients who underwent open reduction showed a CPCHILD II score of 8.6, a CPCHILD III score of 39.4, and a CPCHILD VII of 150.1 points on average. There was no significant difference between the techniques (Table 2).

**Table 2:** CP child score outcome comparisons.

		Mean	Median	Standard deviation	Min	Max	N	CI	*P*-value
CPCHILD II	Open	8.6	6	6.4	3	23	22	2.7	.360
	Closed	10.7	8	8.9	1	40	80	2.0	
CPCHILD III	Open	39.4	45	9.7	14	50	22	4.0	.654
	Closed	40.4	45	9.2	5	55	80	2.0	
CPCHILD VII	Open	150.1	155	16.4	110	170	22	6.8	.244
	Closed	154.7	155	15.9	100	180	80	3.5	

CI = confidence interval, CPCHILD = Caregiver Priorities and Child Health Index of Life with Disabilities Questionnaire, max = maximal, min = minimal, N = sample size.

Furthermore, closed reduction was compared with open reduction in patients presenting Tönnis classification grades III and IV (RMP > 100%), to homogenize the sample and compare only severe cases.

It was observed that open reduction patients had a mean postoperative RMP of 13.8% and a clinical hip abduction of 22.3°. On the other hand, patients who underwent closed reduction had a mean postoperative RMP of 10.5% and an abduction of 20°. Neither parameters showed significant statistical differences (Table 3). Evaluating the VAS for postoperative pain between open and closed reduction groups, an average score of 1.3 and 1.2, respectively, was found. Therefore, both groups are classified in the painless or mild pain category (Table 3).

**Table 3:** Postoperative variables (Tönnis 3 and 4).

		Mean	Median	Standard deviation	CV (%)	Min	Max	N	CI	*P*-value
Reimers after	Open	13.8	0	20.6	150	0	66	12	11.6	.709
	Closed	10.5	0	24.6	235	0	100	17	11.7	
Abduction after	Open	22.3	20	3.9	17	15	30	13	2.1	.299
	Closed	20.0	20	7.1	35	0	30	17	3.4	
Pain VAS after	Open	1.2	0	2.7	225	0	8	10	1.7	.948
	Closed	1.3	0	2.3	180	0	6	15	1.2	

All values are expressed in percentages (Reimers), degrees (abduction), or points (VAS).

VAS = visual analog scale.

Of patients undergoing open hip reduction, 44.8% had Tönnis grades III or IV (*P* = .002). In the closed reduction group, only 17.9% of the patients were classified as Tönnis grades III and IV (Table 4).

**Table 4:** **Hip status outcome comparisons.** *

Hip status (grade)	Open		Closed		*P*-value
	N	%	N	%	
I	10	34.5	40	42.1	.464
II	6	20.7	38	40.0	.057
III	12	41.4	14	14.7	.002
IV	1	3.4	3	3.2	.938

* Total sample number reflects available complete records (124/130).

**Figure 2.**
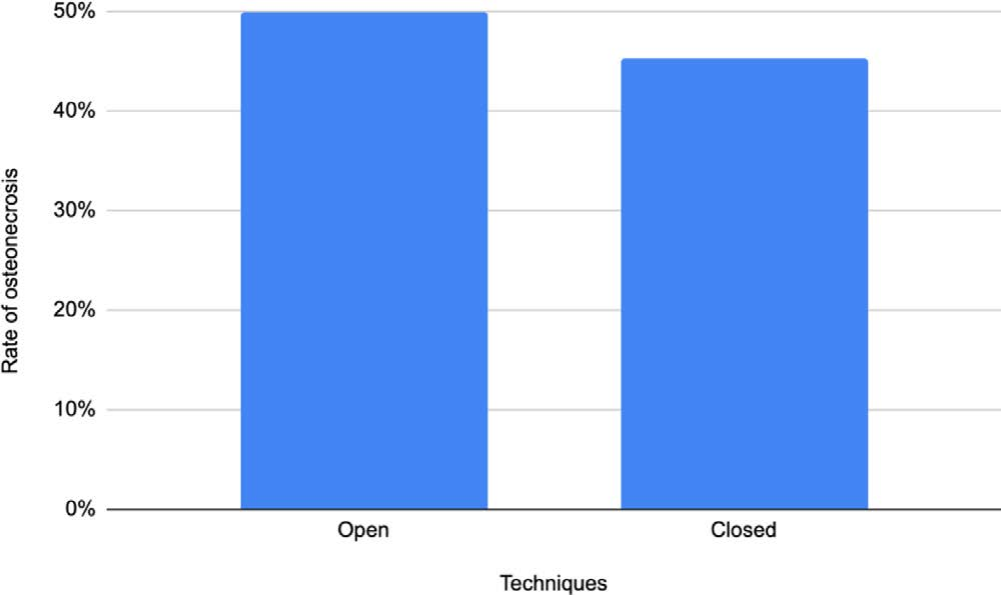
Osteonecrosis rates between techniques.

Figure 2 compares the groups regarding osteonecrosis rate. Osteonecrosis occurred in 50% of the operated hips in the open reduction group, while 45.3% of the hips of the closed reduction group were affected (*P* = .659).

## 4. Discussion

This study compared outcomes of open and closed hip reductions in CP patients undergoing HRS. Both techniques produced similar improvements in radiographic parameters and clinical function and no significant differences were observed in RMP, hip abduction, pain or CPCHILD scores.

Hip dysplasia in patients with CP can lead to pain, contractures, difficulty in hygiene and positioning, pelvic obliquity, scoliosis, skin ulcerations, and fractures, among other complications.^[7,16]^ Hip reconstruction, even in GMFCS III, IV, and V patients, should be performed in cases of subluxation or dislocation. Hip reduction stabilizes the pelvis and prevents joint deterioration, pain, pelvic tilt, and scoliosis.

The main cause of hip dysplasia in CP patients is the spasticity of the adductors and hip flexor muscles, which, being stronger than hip extensors and abductors, creates an imbalance of muscular forces with progressive femoral head extrusion.^[6,17]^ That imbalance, combined with increased anteversion and/or valgus may contribute to the femoral deformity. Noninvasive therapeutic options for hip dysplasia, such as orthoses and physiotherapy, do not appear to change natural history, making surgical treatment the best option.^[7]^

It is important to consider risk factors for postoperative resubluxation and complications, such as avascular necrosis.^[11]^ Sankar et al^[18]^ showed good results with a 16-year follow-up in dislocated hips and CP patients through hip reconstruction with joint capsule opening. They had few complications, promoting a stable and painless hip, improvement in hygiene, absence of pressure ulcer formation, better positioning in the wheelchair, and improved walking in ambulatory patients. On the other hand, Huh et al^[6]^ compared isolated varus derotational osteotomy and varus derotational osteotomy with open hip reduction. Their results showed no statistically significant difference in the rate of recurrent subluxation/dislocation, as well as no significant difference in complication rates between the 2 groups. In our study, the clinical functional analysis of Section II of CPCHILD – which assesses the difficulty level the patient presents in lying down, getting out of bed, transferring from a wheelchair, performing orthostasis, and getting in and out of vehicles – showed low scores in both groups (*P* = .84; Table 3). This result was expected since most of our patients were GMFCS IV and V.

Regarding Section III of CPCHILD – which relates to the degree and frequency of discomfort or pain that the patient experienced while eating, using the bathroom, putting on clothes, transferring from place to place, sitting or lying down – we also found no difference between groups (*P* = .54), but both of them showed higher scores (Table 3). This allows us to conclude that patients did not experience pain or experienced little pain in the postoperative follow-up. Furthermore, we observed that patients in both groups showed satisfactory results related to pain scores, with values of VAS criteria showing results close to 1 (*P* = .94; Table 3).

Mubarak and Wenger^[19]^ treated 18 spastic dislocated or subluxated hips with an average follow-up of 6 years, of which 17 remained anatomically reduced until the last follow-up. A traditional open reduction was done in all cases. In our study, we found that the mean preoperative RMP was 77.4% in patients with open reduction compared to 76% in those who underwent closed reduction (*P* = .801). After the surgical procedure, we observed that the mean RMP was 7.6% and 6.2%, respectively (*P* = .656). These findings suggest that both open and closed reduction, when appropriately selected intraoperatively, lead to similar improvements in hip containment.

McNerney et al^[20]^ performed capsuloplasty if the RMP was >70% or if the femoral head did not fully reduce within the acetabulum (decreased center-edge angle). The authors believe that there may be a 60% redislocation rate if capsuloplasty is not performed, compared to a 3% redislocation rate in patients with a RMP >70% who underwent capsuloplasty. Farcetta et al^[21]^ found that the reducibility test, in the operating room, showing normalization of the Wiberg center-edge angle is a good parameter to decide on the need for open reduction to achieve a concentric reduction. The authors believe that the joint capsule is not a stabilizing factor for the hip in these patients.

Analyzing patients undergoing open hip reduction, 44.8% had grade III or higher status. Furthermore, out of 30 patients with hips with grade III and IV status, 13 (43%) underwent open reduction. We identified the need for stratified evaluation of the sample (hips with grade III and IV status), as there was a higher number of hips that underwent open reduction. However, we did not observe a difference in results between the techniques in this subgroup of patients.

Regarding the age at the time of surgery, Reimers^[22]^ observed better results when surgery was performed before the age of 4. Turker and Lee^[5]^ did not find a correlation between the age at the time of surgery and the postoperative outcome. Although in our study we observed that the average age of patients who underwent open reduction was 116 months (9.6 years), compared to 117.8 months (9.8 years) in the closed reduction group, we did not evaluate the correlation between surgery age and outcomes in each group, which could be done in further studies.

Limiting factors of the study include its retrospective design and the fact that we could not evaluate about 50% of the initially operated sample, which could result in a selection bias. Another factor that can be considered a limitation was the inability to obtain the preoperative quality of life scale (CPCHILD) and the subjectivity of the VAS for pain scale. Furthermore, even though similar results were found between open and closed reductions, the non-randomized retrospective design of the study cannot answer if closed reductions can be performed in all cases, since intraoperative decisions might have led surgeons to conduct an open reduction after attempting and failing a closed reduction. Additionally, no multivariable regression was performed to adjust for potential confounders such as preoperative severity or age.

Despite these limitations, our study has many strengths. First, our data shows that if a concentric reduction is achieved, even in fully dislocated hips, a closed reduction results in satisfactory results. Second, we showed that both techniques lead to good clinical outcomes, a feature that was not analyzed by most of the studies of the literature.^[3,6,8–10,19,20]^ Finally, the number of hips evaluated (130 hips) and the mean follow-up time (49.6 months) were significant to add quality data to the literature and help possible future studies about this subject to define clear guidelines for the treatment of hip dysplasia in CP.

We conclude that open reduction and closed reduction were effective in treating hip dysplasia in patients with CP. There was no statistical difference between the 2 procedures when analyzing clinical and radiographic factors. Due to the retrospective nature of our study, we cannot recommend surgeons to perform closed reductions in all cases, since intraoperative decisions that led surgeons to open reduction when stability was not achieved with closed reduction were not analyzed. Despite that, this study adds evidence that both reduction techniques can lead to satisfactory outcomes when appropriately selected intraoperatively.

Further prospective studies with stratified randomization and multivariable analysis are needed to determine predictors of failure and success to design precise guidelines on selecting between open and closed reduction techniques in CP hip dysplasia.

## Acknowledgments

The authors report no actual or potential conflict of interest with this article. We thank all our subjects for their participation.

## Author contributions

**Conceptualization:** Marcio Vieira Sanches Silva, Bárbara Lívia Corrêa Serafim, Luiz Renato Agrizzi de Angeli, Alexandre Zuccon.

**Data curation:** Marcio Vieira Sanches Silva, Bárbara Lívia Corrêa Serafim, Luiz Renato Agrizzi de Angeli, Alexandre Zuccon.

**Formal analysis:** Marcio Vieira Sanches Silva, Alexandre Zuccon.

**Investigation:** Marcio Vieira Sanches Silva, Alexandre Zuccon.

**Methodology:** Marcio Vieira Sanches Silva, Bárbara Lívia Corrêa Serafim, Luiz Renato Agrizzi de Angeli, Alexandre Zuccon.

**Project administration:** Marcio Vieira Sanches Silva, Alexandre Zuccon.

**Supervision:** Marcio Vieira Sanches Silva, Luiz Renato Agrizzi de Angeli, Alexandre Zuccon.

**Validation:** Marcio Vieira Sanches Silva, Luiz Renato Agrizzi de Angeli, Alexandre Zuccon.

**Visualization:** Marcio Vieira Sanches Silva, Bárbara Lívia Corrêa Serafim, Luiz Renato Agrizzi de Angeli, Alexandre Zuccon.

**Writing – original draft:** Marcio Vieira Sanches Silva, Bárbara Lívia Corrêa Serafim, Alexandre Zuccon.

**Writing – review & editing:** Marcio Vieira Sanches, Bárbara Lívia Corrêa Serafim, Luiz Renato Agrizzi de Angeli, Alexandre Zuccon.

## Supplementary Material


