# Supplementary material for: Surgical treatment of hip dysplasia in cerebral palsy: A retrospective comparison between open and closed reduction
Source: Medicine (Baltimore). 2025 Sep 5;104(36):e44245. doi: 10.1097/MD.0000000000044245 (PMC12419277; doi:10.1097/MD.0000000000044245)
Supplement: Supplementary file 1 [file medi-104-e44245-s001.docx]

**Table 1.** Tonnis classification (12)

| Grade | Criteria |
| --- | --- |
| 1 | Capital femoral epiphysis medial to Perkins line |
| 2 | Capital femoral epiphysis lateral to Perkins line but below the level of the superior acetabular rim |
| 3 | Capital femoral epiphysis at the level of the superior acetabular rim |
| 4 | Capital femoral epiphysis above the level of the superior acetabular rim |
